# Cytometric fingerprints: evaluation of new tools for analyzing microbial community dynamics

**DOI:** 10.3389/fmicb.2014.00273

**Published:** 2014-06-04

**Authors:** Christin Koch, Falk Harnisch, Uwe Schröder, Susann Müller

**Affiliations:** ^1^Department of Environmental Microbiology, UFZ – Helmholtz-Centre for Environmental ResearchLeipzig, Germany; ^2^Institute of Environmental and Sustainable Chemistry, TU BraunschweigBraunschweig, Germany

**Keywords:** microbial flow cytometry, cytometric fingerprinting, microbial fuel cells, electrochemical active microbial biofilms, natural communities, cytometric data analysis, cytometric pattern analysis

## Abstract

Optical characteristics of individual bacterial cells of natural communities can be measured with flow cytometry (FCM) in high throughput. The resulting data are visualized in cytometric histograms. These histograms represent individual cytometric fingerprints of microbial communities, e.g., at certain time points or microenvironmental conditions. Up to now four tools for analyzing the variation in these cytometric fingerprints are available but have not yet been systematically compared regarding application: Dalmatian Plot, Cytometric Histogram Image Comparison (CHIC), Cytometric Barcoding (CyBar), and FlowFP. In this article these tools were evaluated concerning (i) the required experience of the operator in handling cytometric data sets, (ii) the detection level of changes, (iii) time demand for analysis, and (iv) software requirements. As an illustrative example, FCM was used to characterize the microbial community structure of electroactive microbial biofilms. Their cytometric fingerprints were determined, analyzed with all four tools, and correlated to experimental and functional parameters. The source of inoculum (four different types of wastewater samples) showed the strongest influence on the microbial community structure and biofilm performance while the choice of substrate (acetate or lactate) had no significant effect in the present study. All four evaluation tools were found suitable to monitor structural changes of natural microbial communities. The Dalmatian Plot was shown to be most sensitive to operator impact but nevertheless provided an overview on community shifts. CHIC, CyBar, and FlowFP showed less operator dependence and gave highly resolved information on community structure variation on different detection levels. In conclusion, experimental and productivity parameters correlated with the biofilm structures and practical process integration details were available from cytometric fingerprint analysis.

## Introduction

Flow cytometry (FCM) is a high throughput method for analysis of optical characteristics of cells. Its main advantages are the fast analysis, high measuring accuracy, and sensitivity on the single cell level. Thus, FCM is widely applied for biological analysis, especially in medical routine diagnosis and medical research. Compared to that, the application of FCM for the characterization of microbial cells is less common (Web of Knowledge “flow cytometry” 85,039 hits, “flow cytometry bacteria” 3093 hits, 2014/03/17). Especially the characterization of complex microbial communities by FCM, which is termed cytometric fingerprinting, is still rare. Therefore, the following article compares and discusses recently published methods for FCM data analyses of complex microbial communities to expand the application of cytometric fingerprinting.

**Figure 1:**
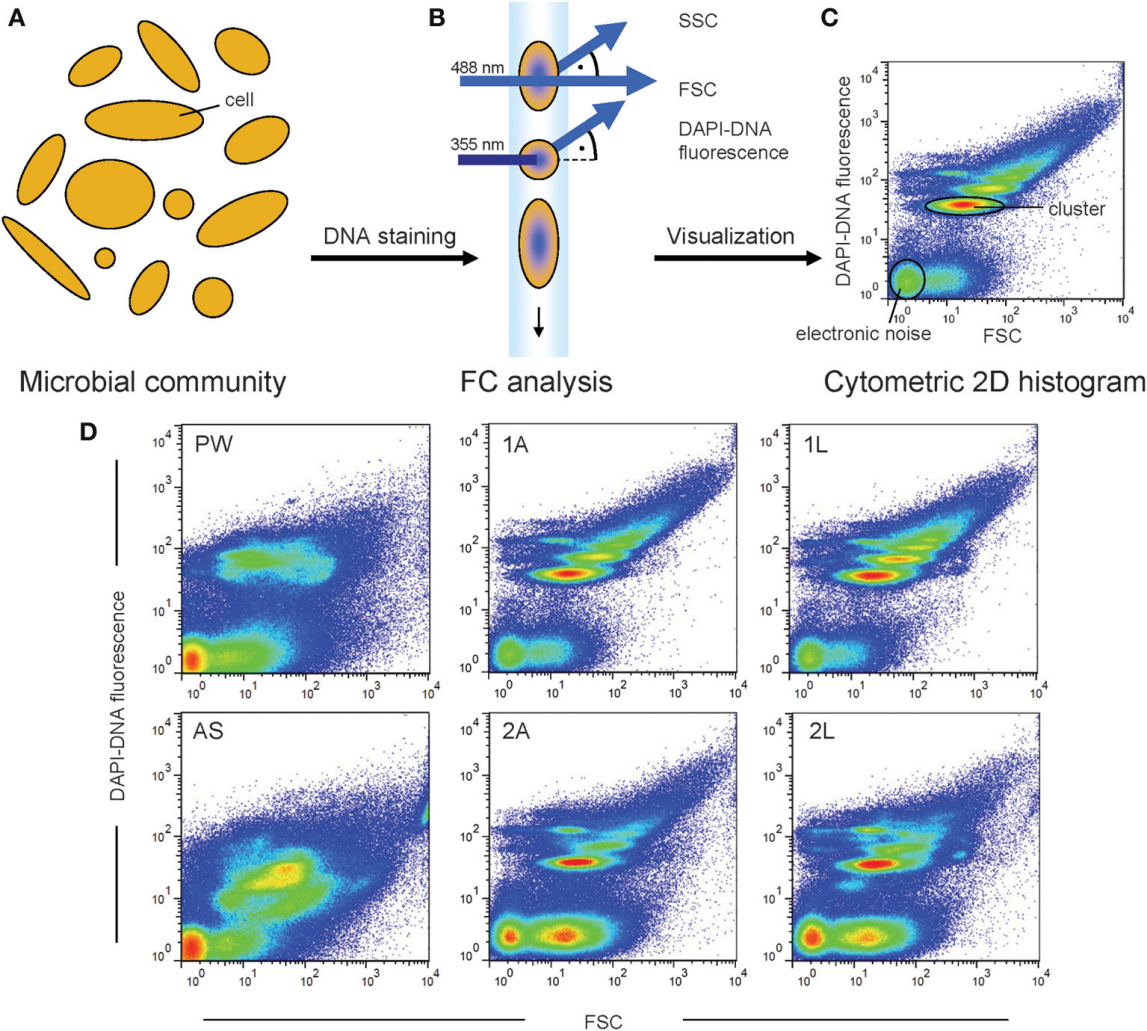
**Natural microbial communities comprise a high diversity of uncharacterized microbial cells (A)**. The individual structure of these microbial communities can be characterized using FCM. The cells are stained with the DNA specific binding molecule DAPI and the cellular characteristics FSC and DAPI-DNA fluorescence can be recorded **(B)**. The cytometric histogram visualizes the measurement of one sample **(C)**. Each virtual cell in the histogram represents the characteristics of a cell regarding the two chosen optical parameters. Therewith, the cytometric histogram can be regarded as cytometric fingerprint of the microbial community structure. Different microbial communities will be characterized by differences in their cytometric fingerprints **(D)**.

The measuring principle of FCM is the following (see also Figure 1): the individual cells of a microbial community are arranged within a liquid stream by hydrodynamic focusing. The cells individually pass a laser beam and thereby their intrinsic properties (cell size, morphology, and granularity) lead to specific interactions with the laser light, including light scattering and fluorescence. Usually, the scattered and refracted light is detected at low angle, i.e., below 2° deviation from the incident light beam that is denominated as Forward Scatter (FSC), or perpendicular to the incident light, denominated as Sideward Scatter (SSC) (Shapiro, 2003) (Figure 1B). Furthermore, fluorescence light can be detected if suitable fluorophores are present. Thus, every cell is represented by an individual set of optical parameters and the acquisition of this set of parameters is performed for every single cell during FCM measurement. In a two dimensional (2D) histogram all cells of a given sample are represented as virtual cells (see Box 1) visualizing the cell's characteristics regarding the chosen optical parameters. Consequently, if cells have very similar optical characteristics clusters of virtual cells will be created in a histogram. These clusters represent subcommunities of a microbial community and thus are of high interest for data analysis, as demonstrated below.

Box 1 **Definitions.**

**Microbial community**: Is the entity of microorganism in a natural sample. It can comprise high diversity, i.e., hundreds of different species regarding phylogeny and function.

**Cell**: The microbial cell is an individual biological unit. It is characterized by optical properties which can be measured using FCM.

**Virtual cell**: The virtual cell represents the cell's characteristics regarding the chosen optical parameters usually in a 2D histogram.

**Cluster**: Virtual cells with similar optical properties. In microbial community analysis a cluster is representative of a microbial subcommunity.

**Segregated data analysis**: Allows a differentiated (or discriminated) analysis of cytometric data sets. It is possible with gate or grid information (see below).

**Gate**: A gate marks a cluster of cells in the histogram that differ from others in their optical properties. It can be defined using one, two, or even more parameters. Methods in microbiology: Dalmatian Plot, CyBar.

**Grid**: The use of a geometrical grid is an alternative to cluster based gating of FCM histograms. Methods in microbiology: Quadrant markers, FlowFP. Image based data analysis using CHIC is also performed based on a geometrical grid.

**Gate template**: Represents the entity of all gates. It is defined by marking all upcoming clusters of one defined experimental series and finally applied to all samples within this experiment.

**Cytometric fingerprint** (= cytometric pattern): It represents the microbial community structure by the number of clusters, the position of these clusters in the histogram, and the number of cells within each cluster.

**Cytometric barcode** (CyBar): Is the variation of the cytometric fingerprint over time or in dependence on experimental factors.

**Operator dependence**: The personal impact on the data evaluation procedure differs between methods, e.g., manual gating vs. automatic grid procedure. A method is defined as operator independent, if a meaningful result can be obtained by using predefined automatic settings. Usually, a cytometric background of the operator is not vital.

Cytometric fingerprinting can be used to detect changes in the structure of microbial communities. If cells disappear or accumulate virtually in certain positions of a 2D-histogramm or alter their optical characteristics these structural community changes become visible by comparing the cytometric fingerprints of two sampling points. A typical measurement for characterizing natural microbial communities includes 250,000 cells and takes about 3 min. Here, FSC and fluorescence are established parameters for characterization (Kleinsteuber et al., 2006; Günther et al., 2009). Thus, as usually most cells show no substantial autofluorescence, it is necessary to use a fluorescence staining labeling all microbial cells. As every microbial cell contains DNA, the use of the highly specific DNA-binding molecule 4′,6-diamidino-2-phenylindole (DAPI) is recommended (Meistrich et al., 1978). However, it should be considered that the effectivity of staining can vary, depending, e.g., on the cell type and state (Müller and Nebe-von-Caron, 2010). When using DAPI not only all cells are stained (and thus can be detected), but also their cellular DNA content can be quantified by the fluorescence intensity. As the cellular DNA content is dependent on cell proliferation and cell division states, environmental alterations causing variations in growth velocity and, therewith, in proliferation activity can easily be detected using FCM (Müller, 2007). A variation of other detectable intrinsic cell properties, e.g., the cell size related distribution, is reflected by the FSC signal. Therefore, the resulting cytometric fingerprint based on FSC and the DAPI-DNA fluorescence in a 2D histogram (Figure 1C) represents the microbial community structure at the point of measurement. This fingerprint is (almost) unique by the number of cell clusters, the position of these clusters in the histogram, and the numbers of cells within each cluster (Koch et al., 2013c).

When looking at various FCM histograms, i.e., respective cytometric fingerprints, differences can be spotted by the naked eye (e.g., Koch et al., 2013c). However, the challenge is to quantify these differences. Therefore, the information of two parameters for each virtual cell has to be transferred to a matrix that is suitable for evaluation. Only then FCM histograms can be exploited for further analysis, e.g., for following dynamics of microbial community structures in response to environmental changes or to compare microbial communities from different origins.

Four methods have recently been applied to analyze changes in the microbial community structure based on cytometric fingerprint measurements: Dalmatian Plot (Bombach et al., 2011), Cytometric Histogram Image Comparison (CHIC, Koch et al., 2013a), Cytometric Barcoding (CyBar, Koch et al., 2013b,c), and FlowFP (Rogers and Holyst, 2009; De Roy et al., 2012). The four methods differ in their analyzing principles and procedures. Therefore, this article will contrast and evaluate the four tools regarding methodical differences. Subsequently, they are assessed toward their ability to resolve variations in cytometric fingerprints. As example, a FCM biological data set resulting from eight electroactive microbial biofilms grown under different substrate and inoculum conditions and being characterized on their performance parameters is evaluated using all methods.

## Methods

### Principles of data analysis

**Figure 2:**
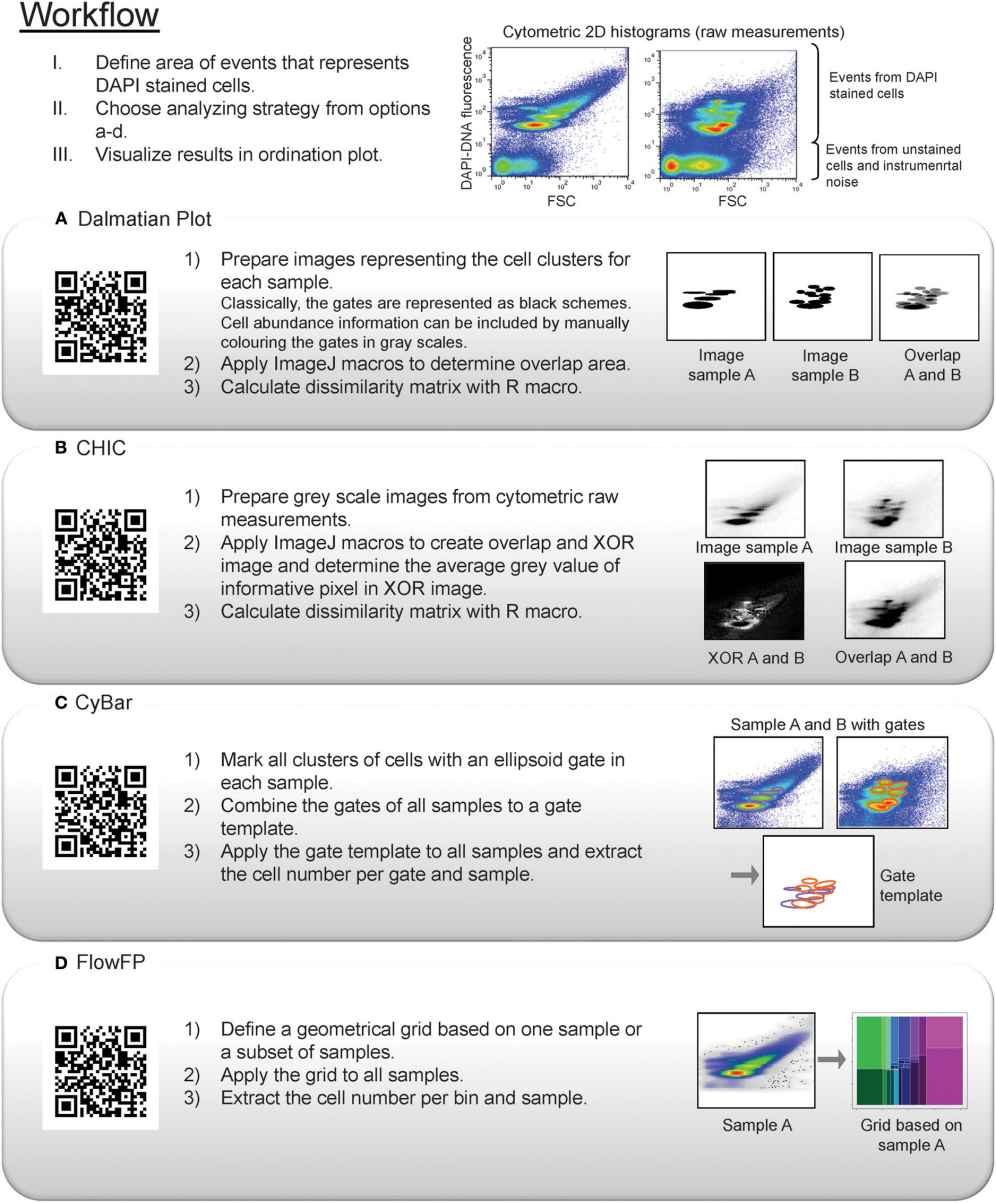
**Workflow for the data analysis procedure of cytometric fingerprints applying Dalmatian Plot, CHIC, CyBar, or FlowFP**. The QR code links to the ready-to-use files for each procedure.

Four methods can be used to evaluate cytometric fingerprint data sets of microbial communities: Dalmatian Plot, CHIC, CyBar, and FlowFP. Their working principles and procedures are explained in the following and summarized in Figure 2.

#### Dalmatian plot

The Dalmatian Plot was first described by Bombach et al. (2011). The name refers to the simplified black-and-white images that are generated during the analyzing procedure. The first step of the procedure is that the most abundant subsets of cells in the 2D histograms are encircled by the operator. This is performed for each plot, i.e., measurement, individually (the gating procedure). The resulting images then represent black blots on a white background for every measurement. Noteworthy, the relative abundance information of the individual clusters is lost when only the black-and-white combination is used, thus representing presence/ absence information. Higher resolved information can be obtained if the cell number is integrated as gray level of the blots (Bombach et al., 2011; Müller et al., 2012). In the second step, the simplified images are processed with image analysis software. This image analysis procedure can be automatically performed using ready-to-use macros (http://www.ufz.de/index.php?en=32660) and the freely available software ImageJ (Schneider et al., 2012). The macros determine the relative area of the gates in each image by counting the number of black pixels. Then, an overlap image for each pair of images is created and the pixel number of overlapping gates determined. Afterwards, the dissimilarity *P_sim_* between each pair of images is calculated based on a modified Jaccard index or distance, respectively (Bombach et al., 2011; Patil et al., 2011). A dissimilarity matrix of all pairs of images under study is automatically created and can be used for statistical analysis and visualization in an ordination plot or cluster analysis, e.g., using R (R Core Team, 2012). All macros and a detailed description of the procedure are available under the QR-Code provided in Figure 2 and the following link http://www.ufz.de/index.php?en=32660.

#### Cytometric histogram image comparison (CHIC)

CHIC is also an image based analysis tool but, in contrast to Dalmatian Plot, does not require an initial manual gating step. The 2D histograms are directly converted to gray scaled images using cytometric software like Summit 3.1 (DakoCytomation). Based on the binary code of the electronic signal acquisition, a histogram resolution in the range of 64, 128, 256, 512, or 1024 channel numbers can usually be chosen and will be reflected in images that will be created from the histograms. Each image will, therefore, have a defined resolution, representing the size of a grid. To evaluate the images the same regular grid should be chosen for all measured samples of a sampling campaign. Images are created from histograms that represent the virtual cells in a linear gray scale intensity representative for the relative abundance of cells per grid. Using ImageJ the cytometric images are compared to each other. This comparison is based on two mathematical computations, which are performed on a pixel to pixel basis for every pair of images. The exclusive disjunction function creates an XOR image of two cytometric images while the second algorithm produces their overlap. Subsequently, the average gray value per informative pixel, i.e., pixel resulting from virtual cells, is calculated using the sum of all pixel values from the XOR image and the number of informative pixels from the overlap image. The average gray value can directly be used as dissimilarity value *P_sim_* for each pair of images and a dissimilarity matrix of all pairs of images is automatically created. It can be used in the same way as with Dalmatian Plot for statistical analysis and visualization. All macros are available in (Koch et al., 2013a) and can also be found under the QR-Code provided in Figure 2 and the following link http://www.ufz.de/index.php?en=32736.

#### Cytometric barcoding (CyBar)

CyBar performs a segregated analysis of cytometric histograms without any image analysis step. In this procedure, like in most analyzing procedures in FCM, an operator dependent, and thus experience based gating step has to be performed. Every cluster of cells in a histogram is marked with a gate. The individual gates of each sample are combined to one gate template for a data set. Such a gate template can comprise up to 30 gates and more when natural microbial community data sets are explored (Koch et al., 2013c). The gate template serves then as a mask which is applied to all samples of the data set. The cell abundances in each gate are easily extracted for all samples. Therewith, the abundance variation per gate can directly be compared between samples of different treatments or over a time course. The direct comparison of cell abundance variations between gates with high and low cell numbers is facilitated by data normalization. The dynamic variations of abundances per gate are then visualized in form of a barcode like heat map, the CyBar plot (performed in R, Figure S1). The CyBar plot allows identifying stable or highly fluctuating subsets of cells. In this way, a segregated analysis of individual cell cluster responses is possible in addition to the general trend interpretation analysis which was already provided by Dalmatian Plot and CHIC. Moreover, index subcommunities can be identified, i.e., potential functions assigned to clusters of cells by correlation analysis, and sorted for further analysis. A detailed step by step procedure and ready-to-use macros for the CyBar procedure are provided in Koch et al. (2013c) and were recently published as R package on the Bioconductor platform (www.bioconductor.com as flowCyBar, http://www.bioconductor.org/packages/devel/bioc/html/flowCyBar.html). The link is also available under the QR-Code provided in Figure 2.

#### FlowFP

FlowFP (Rogers and Holyst, 2009) is a software package of the Bioconductor platform (Gentleman et al., 2004). Thereby, the complete analyzing procedure can be performed in R. FlowFP was first developed for handling FCM data sets for medical research, but was recently also successfully applied to a microbiological data set (De Roy et al., 2012). The FlowFP analyzing procedure does not require an image analysis step or any manual gating decision but works on the basis of a geometrical grid. The application uses a probability distribution function to define two regions of the FCM histogram that contain an equal number of cells. These regions are considered as bins and further partitioned with the identical probability distribution function creating equal sub-bins with identical virtual cell numbers. This procedure is repeated for every bin, based on a predefined number of recursions. The result is a geometrical grid with fixed numbers and positions of bins. Consequently, bins in regions with high abundance of virtual cells are smaller compared to those covering regions with low cell abundance. The grid can be built based on one sample or a set of samples. Subsequently, the computed grid serves as a mask which is applied to a complete data set. The number of cells per bin is extracted and stable and fluctuating bins can be identified. Therewith, segregated dynamics within microbial communities can be investigated as well as similarity analyses performed. For extensive information on FlowFP see Rogers and Holyst (2009) and (http://www.bioconductor.org/packages/release/bioc/html/flowFP.html). A ready-to-use macro for the application of FlowFP to microbial cytometric fingerprints based on FSC and DAPI-DNA fluorescence (application example below) was created and is available under the QR-Code provided in Figure 2 and the following link http://www.ufz.de/index.php?en=32738.

### Experimental setup of application example

The applicability of the above described four approaches for FCM data analyses was tested with a real biological data set. Anodic mixed culture derived electroactive microbial biofilms were characterized electrochemically and using FCM. Two important variables for the formation and performance of electroactive microbial biofilms were studied: (i) the inoculum, i.e., the source of the bacterial diversity, and (ii) the microbial substrate, i.e., the electron donor and carbon source. The outcome gained from the FCM data analysis was further evaluated using productivity parameters, i.e., biomass formation, maximum geometric current density (*j*_max_), and coulombic efficiency (*CE*).

#### Electrochemical measurements

All electrochemical experiments were carried out under potentiostatic control, using one-chamber three-necked-flasks (250 mL) with a three electrode arrangement consisting of the working electrode (projected surface area: 8.00 cm^2^), Ag/AgCl reference electrode (saturated KCl, Sensortechnik Meinsberg, Germany, 0.195 V vs. SHE), and counter electrode. The working and counter electrodes used throughout this study were graphite rods (CP-Graphite GmbH, Germany). The experiments were conducted with a Potentiostat/Galvanostat Model VMP3 (BioLogic Science Instruments, France), equipped with 12 independent potentiostat channels. The current density (*j_max_*) is reported per projected surface area and denominated as “geometric current density.” All experiments were conducted under anoxic conditions at 35°C.

Four types of wastewater served as microbial inoculum, i.e., primary wastewater (PW), activated sludge (AS), primary sludge (PS), and secondary sludge (SS). The wastewater samples were collected from the wastewater treatment plant Steinhof, Braunschweig (Germany). The growth medium was prepared as reported by Kim et al. (2005). In order to ensure anaerobic conditions it was purged with nitrogen for 30 min before use. Sodium acetate (10 mM) or sodium lactate (10 mM) served as substrates in the growth medium. An overview on sample denomination, source of microbial inoculum, and substrate choice is given in Table 1.

**Table 1:** **Overview on source of microbial inoculum and substrate for mixed culture derived microbial biofilm experiments and derived sample denomination**.

**Source of inoculum**	**Substrate**	**Sample denomination**
Primary wastewater (PW)	Acetate	1A
Primary wastewater (PW)	Lactate	1L
Activated sludge (AS)	Acetate	2A
Activated sludge (AS)	Lactate	2L
Primary sludge (PS)	Acetate	3A
Primary sludge (PS)	Lactate	3L
Secondary sludge (SS)	Acetate	4A
Secondary sludge (SS)	Lactate	4L

The biofilm formation procedure was followed as described by Liu et al. (2008) in fed-batch experiments. 200 mL of the stirred growth medium were inoculated with 10 mL microbial inoculum. A constant potential of +0.2 V (vs. Ag/AgCl) was applied to the working electrode to facilitate the biofilm formation. The biofilm growth was monitored by measuring the bioelectrocatalytic oxidation current. After substrate exhaustion (determined by HPLC and decrease in oxidation current) the bacterial medium was replenished using fresh solution.

Cyclic voltammetry (CV) was performed during turnover conditions in accordance with previous studies, e.g., Fricke et al. (2008), Srikanth et al. (2008). Potentials were applied from −500 to +300 mV (vs. Ag/AgCl) at a scan rate of 1 mV s^−1^ with continuous monitoring of the current response (Fricke et al., 2008; Srikanth et al., 2008). The total coulombic efficiency (*CE*) was calculated by integrating the current over time and using the acetate respectively lactate consumption data. The electrochemical experiments were carried out three times and the biofilms of the third performance used for cytometric analysis. Original data are shown in Table S1.

#### Analysis of chemical data

The substrate consumption was assessed using HPLC. The HPLC (Spectrasystem P400, FINNIGAN Surveyor RI Plus detector, Fisher Scientific, Germany) was equipped with a Rezex HyperREZ XP Carbohydrate H+ 8 μm column. The chromatograms were recorded at room temperature with 0.005 N sulphuric acid as eluent. Biomass was determined in triplicates as dry weight as described in Patil et al. (2011).

#### Flow cytometry

The biofilm samples were fixed with 10% sodium azide and prepared for cytometric analysis as described in Patil et al. (2011). The sample preparation included washing steps to remove the fixative, separation of the cells by vortex and sonication, and staining using a two-step procedure. The first step is 20 min incubation with a solution containing citric acid and Tween20 to facilitate dye penetration and binding. Afterwards, the samples are incubated with the DAPI staining solution (0.68 μM) for at least 60 min.

The flow cytometric measurements were carried out as described before Patil et al. (2011). A MoFlo cell sorter (DakoCytomation, USA) equipped with two lasers [488 nm and ML-UV (333–365 nm)] was used to analyze FSC, SSC (trigger signal), and DAPI-DNA fluorescence. Fluorescent beads were used to align the instrument: yellow-green fluorescent microspheres (2 μm, FluoSpheres (505/515), Molecular Probes, cat. no. F-8827), blue fluorescent microspheres (1 μm, FluoSpheres (350/440), Molecular Probes, cat. no. F-8815), bright blue Fluoresbrite carboxylate microspheres (0.5 μm (360/407), Polysciences, cat. no. 18339-10). Data acquisition was performed with the Summit v.4.3 software (DakoCytomation, USA).

#### Data analysis procedure

The cytometric measurements of the obtained eight biofilms were analyzed with Dalmatian Plot, CHIC, CyBar, and FlowFP following the above described standard procedures and using the provided macros.

For the Dalmatian Plot the cytometric measurements were converted to simplified black-and-white images which represented the cytometric fingerprint with 5–11 black gates in each image, thus giving equal priority to all emerging clusters independent of their cell abundance. CHIC analysis was performed using a 128 channel resolution and the provided gray scale of the standard procedure. For CyBar, a gate template was constructed consisting of 20 gates. The FlowFP analysis was performed using the samples 1A and 1L to compute the grid based on 5 recursions.

#### Statistics

Statistical analysis was performed with R using the functions “metaMDS,” “envfit,” and “procrustes” from the package vegan (Oksanen et al., 2012). Correlation analysis was based on 999 permutations.

## Results and discussion

### Comparison of tools for microbial community analysis

The four methods Dalmatian Plot, CHIC, CyBar, and Flow FP are available to follow variations in microbial community structures based on cytometric fingerprinting (Figure 2). Here, all four were applied to analyze the same biological data set resulting from eight electroactive microbial biofilms. First, the four tools are assessed regarding methodical differences (see also Table 2). Then, they are compared toward their ability to resolve specific variations in the microbial community structures and interpret community behavior in general and biofilm performance in particular.

**Table 2:** **Comparison of the cytometric fingerprint evaluation tools**.

	**Dalmation plot**	**CHIC**	**CyBar**	**FlowFP**
Outcome	Dissimilarity matrix	Dissimilarity matrix	Matrix with cell numbers per gate for all samples, CyBar plot	Matrix with cell numbers per bin for all samples
Software requirements	Cytometric software, Irfan-View, Paint, ImageJ, R	Cytometric software, ImageJ, R	Cytometric software, R	R
Detection level of changes	Whole community	Whole community	Individual gate	Individual bin
Advantages	Simple, trend interpretation analysis	Operator independent, fast, trend interpretation analysis	Segregated analysis of subcommunity dynamics in addition to trend interpretation analysis, matrix can be used for subcommunity sorting	Operator independent, segregated analysis of dynamics in bins in addition to trend interpretation analysis
Disadvantage	Experience based gating procedure, time consuming	Conversion of histogram to image	Experience based gating procedure	Biological subcommunities are not represented by binning procedure

#### Impact of operator

The four methods require different operator expertise with cytometric fingerprint analysis. So far, none of the analysis protocols can be used as “one-click-method.”

The Dalmatian Plot and CyBar procedure require gating decisions by the operator at the beginning of the analyzing procedure. Gating decisions are individual and also strongly depend on the individual pre-experience with cytometric data analysis. For instance, while one operator defines a smaller number of bigger gates covering clusters of cells in a 2D histogram another operator will use a higher number but smaller gates analyzing the same data set. In general, the gates have to reflect the biological relevant clusters of cells in the histograms and should cover the majority of all virtual cells to allow reasonable interpretation. Histogram visualization using different graph types like pseudocolor, contour, or density plot helps to identify these clusters of cells.

The CHIC procedure is performed without any gating decisions as the complete histogram is converted to an image file. Therewith, the analysis procedure with CHIC is not operator dependent and always leads to the same result matrix. Nevertheless, the CHIC procedure includes adjustment options like the histogram resolution (Koch et al., 2013a). With these adjustments the sensitivity of the analysis can be improved to meet different requirements depending, e.g., on the individual experimental settings, number of recorded cells, or diversity of the microbial community. The FlowFP procedure also works without individual gating decisions, thus, could be performed without any operator impact. Nevertheless, the FlowFP procedure requires the computation of the grid that serves as mask to analyze the data. The definition of the grid is very flexible and certainly influences the analysis result. The sample choice for defining the grid should reflect the diversity of patterns to be analyzed in a data set. By that and an adequate number of recursions it is ensured that regions of high and low virtual cell density are equally covered in all samples and changes in the community structure can reliably be detected.

#### Detection level of differences

All four approaches result in a mathematical matrix, which finally allows visualizing dissimilarities between samples and thus structures of microbial communities.

For Dalmatian Plot and CHIC this matrix represents a dissimilarity matrix which results from the pair wise comparison of all histograms. Each comparison results in a dissimilarity value between two given samples. The value represents the overall dissimilarity for each pair and does not enable to retrieve information about the source of dissimilarity. The dissimilarity can either result from only one cluster of cells showing a strong change in abundance between two samples or from several smaller changes in more than one cluster. However, it is often of high interest to know details about the changes in the microbial community structure. Segregated analysis of subcommunity dynamics in addition to trend interpretation analysis is only possible with CyBar and FlowFP. Both enable the detection of structural community changes down to the individual gate or bin, respectively.

For CyBar the gate template based analysis allows directly visualizing the differences in abundance of each subset of virtual cells using the CyBar plot (see also Figure S1). Therewith, it is easily possible to allocate changes in community structure to certain clusters that rise or reduce their cell numbers. In addition, clusters with similar or opposite response can be identified. The gate template has another advantage. It can directly be used as template for cell sorting; thus, cell clusters of interest can be manually separated from the whole community and afterwards further investigated based on genomic or proteomic techniques (Jahn et al., 2013; Koch et al., 2013c).

FlowFP also allows identifying bins which are stable or show a high variability. Due to the principle of probability binning with equal numbers of cells per bin, regions of very high cell density will result in several smaller rectangular bins in the two dimensional plot. Thus, changes will most often not be restricted to one single bin but affect a number of neighboring bins. However, a bin is an artificial classification based on cell number distribution and independent of potential biological subcommunities. Therefore, it does not mark biological subsets of cells making cell sorting and cell cluster based interpretation, e.g., regarding proliferation changes, more difficult.

As a result of the individual detection levels of differences, the interpretation depth is also limited dependent on the chosen analysis. The structural changes in the microbial community are a response to changes in environmental or experimental parameters. Using statistical analysis the overall change between samples can be correlated with the experimental variables. This general trend interpretation is possible for all four techniques. In addition, the segregated analysis of CyBar and Flow FP allows individual gate or bin correlations, thus the identification of functional subcommunities.

### Application example: cytometric community analysis of electroactive microbial biofilms

The electroactive microbial biofilms were formed at solid carbon electrodes which served as anode, i.e., electron acceptor, using a standard bioelectrochemical three electrode setup. The potential at the anode was constant at 0.2 V (vs. Ag/AgCl). Microorganisms performing extracellular electron transfer (EET), i.e., possessing the ability to transfer electrons to solid terminal acceptors, can oxidize the provided substrates (acetate or lactate) to CO_2_ and H^+^. The resulting EET is measured as flow of electric current. The microbial community structure of the electroactive biofilms was determined using cytometric fingerprinting as method of choice (vide supra) and its performance characterized by electrochemical and biological parameters. Here, the following parameters were used (i) the maximum geometric current density, *j_max_*, which is the maximum number of transferred electrons per second per projected surface area of the electrode, (ii) the Coulombic efficiency, *CE*, which is a measure of the electron transfer to the electrode per electron released by substrate oxidation reaction, and (iii) the biomass of the biofilm. For more details on electroactive microbial communities and their technological potential, e.g., Harnisch and Schröder (2010); Logan and Rabaey (2012); Rabaey and Rozendal (2010). In the current experiment two important variables for their formation and performance were studied: the types of inoculum and substrate.

**Figure 3:**
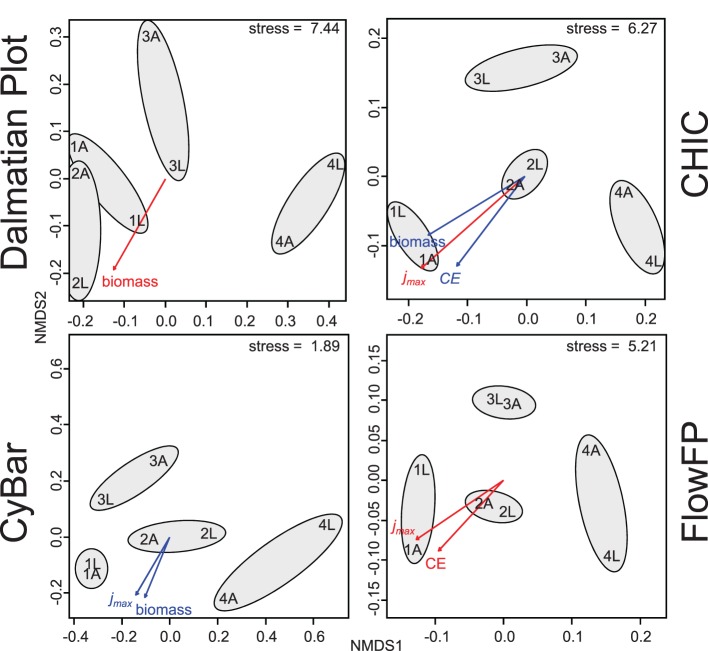
**Dissimilarity analysis results of cytometric fingerprints of electroactive biofilms**. The cytometric fingerprints of eight biofilms were recorded and analyzed with Dalmatian Plot, CHIC, CyBar, and FlowFP. The samples are arranged according to their dissimilarity in the NMDS plots and the gray circles indicate samples with the same inoculum. For further sample denomination see Table 1. The productivity parameters maximum geometric current density (*j*_max_), coulombic efficiency (*CE)*, and biomass of the biofilm were correlated to the cytometric results and significant correlations are displayed with *p* = 0.05 in red and *p* = 0.1 in blue.

The inoculum determines the microbial diversity, i.e., the present species, in each setup that will have the chance to colonize the electrode. The inoculum samples were collected from four basins of a local wastewater treatment plant (PW, AS, PS, SS—see Table 1). As such basins provide different environmental conditions (e.g., carbon sources, oxygen levels, flow through rates), they are generally known to contain different microbial communities (Günther et al., 2012). The choice of substrate, i.e., the electron donor, determines the growth activity of those microorganisms in a bioelectrochemical cell, being able to utilize the substrate either directly by EET or indirectly as part of a food web, e.g., by fermentation. Therewith, the biofilm structure at the anode results from an electrochemically driven selection. Information on the structure of a microbial community in an electroactive biofilm may bare the potential to understand and predict its performance parameters. Therefore, the electroactive microbial biofilms were investigated using cytometric fingerprinting. The obtained histograms were analyzed with Dalmatian Plot, CHIC, CyBar, and Flow FP and the results visualized using non-metric multidimensional scaling (NMDS) plots (Figure 3). The FCM results were furthermore correlated to the bulk measurements of performance parameters in order to reveal structure-function relationships of biofilm productivity and microbial community (see Günther et al., 2012, for comparison).

The results of the dissimilarity analyses of the microbial biofilms were comparable for all four methods. From all plots in Figure 3 it is evident that the origin of the bacterial community, i.e., the source of inoculum, had a major influence on the community structure of the biofilms. The biofilms resulting from the same inoculum were more similar to each other based on their cytometric fingerprints although different substrates were provided. In addition, it has to be mentioned that the cytometric fingerprints of the biofilms were differing from those of the microbial inocula (Figure 1 and Figure S2) emphasizing the electrochemically driven selection. The substrate sources, acetate and lactate, were also found to cause differences in the microbial community structures between the biofilms, but the inoculum was the major determinate.

Two out of eight biofilms, 1A and 1L, with the origin from primary wastewater showed a high electrochemical performance in terms of *j*_max_ and *CE* (for comparison, e.g., Harnisch and Schröder, 2010; Pant et al., 2010). They reached the highest current densities of 659 μA cm^−2^ and 494 μA cm^−2^, and the highest Coloumbic efficiencies of 94% and 25%, respectively, accompanied by high biomass formation [3.68 (±0.76) and 4.17 (±0.28) mg cm^−2^]. Both biofilms showed a comparable maximum performance, but only the biofilm 1A used 94% of the consumed substrate (acetate) for the current production. This is in line with literature, as high performing electroactive microorganisms like *Geobacter* can directly utilize acetate, but not lactate (Speers and Reguera, 2012). In former studies, high performing biofilms were characterized on their electrochemical behavior and possessed properties similar to previous mixed culture derived biofilms that were dominated by *Geobacteraceae* (Torres et al., 2009; Harnisch et al., 2011; Patil et al., 2011). The second best performing pair of biofilms was 2A and 2L resulting from the activated sludge inoculum. Their cytometric fingerprints showed a higher similarity to 1A and 1L than the other biofilms, which was also reflected by the plots in Figure 3. The more the cytometric fingerprints of the biofilms deviated from 1A and 1L the less bioelectrochemically productive were they, along with lower biomass formation [1.21 (±0.14) to 2.50 (±1.09) mg cm^−2^]. This interrelationship between the origin of the community, the resulting biofilm formation, and its productivity can clearly be retrieved from the cytometric data sets and is emphasized by correlation analysis (Figure 3).

From a biological perspective, the microorganisms with the highest capacity for establishing electroactive microbial biofilms seemed preferentially present in the primary wastewater. Primary wastewater is a highly variable habitat which is characterized by numerous organic substances as it is the first basin in the wastewater treatment flow that contains high amounts of C- and N-species. In contrast to the various wastewater basins, the experimental setup offered a highly specialized niche. As expected, the originally highly diverse structure of the primary wastewater community decreased and only a small number of specialists formed the electroactive biofilm. The structural and functional differences of the biofilms in the different experimental setups were well recognized by all four tools (Figure 3 and discussion below) and could be used as basis for further ecological interpretation (Box 2). From an engineering perspective, these results can be of highest value for planning and implementation of bioelectrochemical setups into wastewater treatment plants (Lovley, 2006; Rabaey et al., 2010). The high costs and risks for empirical testing can be reduced using FCM in combination with correlation analysis beforehand as exemplarily demonstrated in the application example.

Box 2 **Ecological interpretation of cytometric data sets.**

In macro- as well as in microbiology biodiversity indices help to investigate large data sets derived from community analysis on respective community characteristics and response to environmental changes (Purvis and Hector, 2000; Prosser et al., 2007; Marzorati et al., 2008). However, specific values for these indices defining a community, e.g., as divers, healthy, or functional are missing (Purvis and Hector, 2000; Read et al., 2011). As demonstrated in the following on the example of CHIC respective cytometric diversity indices can be derived from the cytometric fingerprint. Yet, further comprehensive data set analyses are needed for benchmarking them.

Range-weighted richness (*Rr*)

The *Rr* value represents the number of detected distinct individuals, *N_i_*, in relation to the total number of distinct individuals, *N*_all_. As in FCM individuals are represented by virtual cells in histogram *Rr* can be calculated from the cut images (see CHIC procedure) according to equation 1:

(1) $$Rr\;=\;\frac{N_{i}}{N_{all}}$$

with *N_i_* being the number of pixels containing virtual cell information (being not white) and *N_all_* the number of all pixels of the cytometric image (including informative and non-informative, thus white, pixels).

Structural organization (*So*)

*So* describes the relative difference in abundance of distinct individuals, *P_ij_*, and the average abundance of all distinct individuals, *P*_average_ (see Marzorati et al., 2008). Thus, in cytometric fingerprints the single pixel value represents *P_ij_* and the average pixel intensity of all informative pixels represents *P*_average_ as follows:

(2) $$So\;=\;\frac{\sum_{i;j}\left|P_{ij}-P_{average}\right|}{N_{i}}$$

with *Ni* being the number of pixel bearing virtual cell information (being not white).

Dynamics (*Dy, Da*)

Dynamics describe the degree of variation within a microbial community over time. *Dy* describes the average dissimilarity of consecutive samples and *Da* the average dissimilarities of all samples. In FCM all pairwise dissimilarity values are given in the CHIC result matrix (see Methods as well as Koch et al., 2013a) and can be used to calculate both indices.

Application example

The above described cytometric diversity indices *Rr* and *So* were calculated from the cytometric fingerprint data set of the application example. As the dynamic development of the biofilm was not monitored *Da* and *Dy* were not assessed.

The experimental setup offered a narrow ecological niche as only a single substrate (electron donor) and an electrode as solid electron acceptor were provided. As only a small number of microbial specialists can occupy this niche a reduced number of different individuals were expected. Consequently, these specialists will increase their numbers stronger than other individuals which are less adapted. This expected adaptation is reflected by the cytometric fingerprints of the biofilms. The highest specialization is detected in the PW derived biofilms grown on acetate and lactate, as represented by the lowest range-weighted richness [*Rr* = 0.43 (1A) and *Rr* = 0.46 (1L)] and high structural organization [*So* = 39.3 (1A) and *So* = 43.0 (1L)]. These biofilms were also the most productive ones in terms of current production and efficiency (see Table S1). For results of all biofilms see Table S3.

### Comparability of results

The dissimilarity analysis results of the application example show a high resemblance for all four tools (Figure 3). CyBar, FlowFP, and CHIC show highest similarity in the NMDS plots, which was also supported by procrustes analysis (Table S2), a mathematical procedure to compare ordination results. Possible reasons for the higher deviation of the Dalmatian Plot are (i) that this method is based on individual gating per sample in contrast to the global template (gate, respectively grid), used for the other tools, as well as (ii) that no cell abundance information is exploited (by only using the black-and-white, i.e., presence-absence information).

The correlation of FCM data with the productivity parameters using all four tools showed similar dependencies. Commonly, biofilms derived from primary wastewater and activated sludge resulted in the best and second best performance regarding biomass, *j_max_*, and *CE*. Nevertheless, individual differences on the methods were found. Whereas Dalmatian Plot identified only biomass as significant correlation parameter (*p* = 0.05) two significant correlation parameters were found with CyBar (*j_max_* and biomass, *p* = 0.1) and FlowFP (*j_max_* and CE, *p* = 0.05). With CHIC significant correlations were found for *j_max_* (*p* = 0.05), biomass, and CE (*p* = 0.1).

The major focus for the application example was the detection of trends in community structure variation. This was achieved by using all tools. CHIC and FlowFP offered the fastest analyzing procedures for this purpose including high reproducibility. If a further segregated analysis of subcommunity dynamics, i.e., the contribution of individual subcommunities to the overall change, is aimed, CyBar provided the best option (Figure S1).

### From the detection of changes to biodiversity indices

New cytometric fingerprinting methods allow changes in microbial community structures to be followed. Independent of the different motivations for monitoring microbial communities different information can be derived. One of the prime options is monitoring community stability, which is, e.g., needed for the evaluation of drinking water quality (De Roy et al., 2012; Hammes et al., 2012; Prest et al., 2013). Here, a continuous and fast analyzing procedure is required allowing an instantaneous statement on the (in)stability. As was shown in the current study, CHIC and FlowFP are best suited tools for analyzing FCM-data sets for this kind of application. A more detailed insight into the community changes is given with the second option, the segregated analysis of microbial subcommunity changes. It allows identifying individual contributions of subcommunities to an overall process as, e.g., found in the different basins of a wastewater treatment plant (Günther et al., 2012). Here, CyBar is the best choice for the cytometric fingerprint analysis.

Furthermore, biodiversity measures, which are well established in macroecology theory and that were already applied to taxonomy based microbial data sets (e.g., Purvis and Hector, 2000; Prosser et al., 2007; Marzorati et al., 2008), can also be derived from the cytometric fingerprints. This has been predicted by Wang et al. (2010) but until now no mathematical implementation was shown. Box 2 gives a first approach on how the cytometric diversity indices (i) range-weighted richness, (ii) structural organization, and (iii) dynamics of microbial communities can be calculated from cytometric fingerprints. Henceforth, these indices can help to derive further community characteristics and investigate underlying ecological principles for microbial community behavior.

## Conclusion

All four analysis tools (CHIC, Dalmatian Plot, CyBar, and FlowFP) allow deriving further information of FCM fingerprints. As the tools differ in their principles of data analysis, their detection level of changes differs as well. As FCM fingerprints can sensitively resolve dynamics in microbial communities with high throughput and at low costs, their application for monitoring managed microbial systems (e.g., biotechnology, energy production, drinking water supply) as well as natural environments is highly recommended. Thereby, microbial FCM can be flexibly expanded with advanced -omics techniques in scientific research as well as with practical implementations (Koch et al., 2014a,b).

### Conflict of interest statement

The authors declare that the research was conducted in the absence of any commercial or financial relationships that could be construed as a potential conflict of interest.
