# Supplementary material for: Cytometric fingerprints: evaluation of new tools for analyzing microbial community dynamics
Source: Front Microbiol. 2014 Jun 4;5:273. doi: 10.3389/fmicb.2014.00273 (PMC4044693; doi:10.3389/fmicb.2014.00273)
Supplement: Supplementary file 1 [file DataSheet1.DOC]

**Supplementary Information**

**Cytometric fingerprints: evaluation of new tools for analyzing microbial community dynamics**

Christin Koch, Falk Harnisch, Uwe Schröder, Susann Müller

| **Supplementary File** | **Title** | **Page** |
| --- | --- | --- |
| Supplementary Figure 1 | CyBar plot for cytometric data set of the mixed culture derived electroactive microbial biofilms | SI 2 |
| Supplementary Figure 2 | Cytometric pattern of inoculum and biofilm samples | SI 3 |
| Supplementary Table 1 | Electrochemical and experimental data | SI 4 |
| Supplementary Table 2 | Procrustes sum of squares | SI 4 |
| Supplementary Table 3 | Results for *Rr* and *So* values | SI 5 |

**Supplementary Figures**


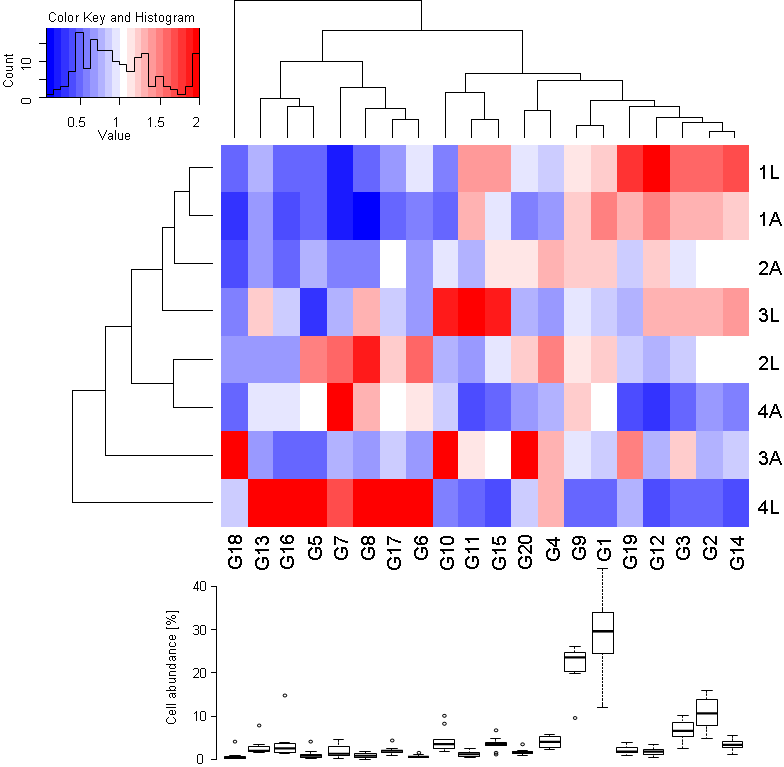


**Supplementary Figure 1**: CyBar plot for the cytometric data set of the mixed culture derived electroactive microbial biofilms. The relative variations of abundances per gate are visualized as a heat map. Every horizontal line represents one sample (1A, 1L to 4A, 4L as described in the article). The relative abundances of each gate (G1 to G20) per sample are shown.

From this form of visualization it becomes obvious that sample 1L and 1A are highly similar regarding cell numbers in nearly all gates. In the CyBar plot the gates G1-G3, G9, G12, G14 and G19 represent the highest while other gates (e.g. G18 or G6) show lowest cell abundances for these two samples. In accordance with the ordination plot in Figure 3, the sample 4L shows the lowest similarity to the other samples.

In the lower part of the figure, the cell abundance per gate relative to all cells is shown. This allows identifying G1 as the gate with the highest cell abundance followed by G9, G2 and G3.

**Supplementary Figure 2**: Cytometric pattern of inoculum and biofilm samples. The sample denomination is according to Table 2. The microbial community structure of the electroactive microbial biofilms deviated from the original wastewater inocula.

**Supplementary Tables**

**Supplementary Table 1**: Electrochemical and experimental data.

Experimental parameters and electrochemical data for anodic mixed culture derived electroactive microbial biofilms. The data represent average values from three independent biological replicates. See manuscript for experimental details and sample denomination. The current density (*j*_max_) is reported as “geometric current density” normalized to the electrode surface area, as well as the biomass.

| sample | *j*_max_ /  µA cm^-2^ | *CE* /  % | biomass/  mg cm^-2^ | substrate  lactate acetate | | inoculum  PW AS PS SS | | | |
| --- | --- | --- | --- | --- | --- | --- | --- | --- | --- |
| 1L | 494 (±146) | 24.7 (±12.1) | 4.17 (±0.28) | 1 | 0 | 1 | 0 | 0 | 0 |
| 2L | 171 (±95) | 5.6 (±4.6) | 3.84 (±1.54) | 1 | 0 | 0 | 1 | 0 | 0 |
| 3L | 147 (±177) | 1.5 (±0.9) | 2.50 (±1.09) | 1 | 0 | 0 | 0 | 1 | 0 |
| 4L | 32 (±35) | 1.0 (±0.6) | 1.21 (±0.14) | 1 | 0 | 0 | 0 | 0 | 1 |
| 1A | 659 (±112) | 94.4 (±1.2) | 3.68 (±0.76) | 0 | 1 | 1 | 0 | 0 | 0 |
| 2A | 240 (±274) | 24.6 (±13.7) | 2.45 (±0.23) | 0 | 1 | 0 | 1 | 0 | 0 |
| 3A | 50 (±24) | 7.5 (±5.2) | 1.35 (±0.16) | 0 | 1 | 0 | 0 | 1 | 0 |
| 4A | 75 (±141) | 5.5 (±3.5) | 2.45 (±0.84) | 0 | 1 | 0 | 0 | 0 | 1 |

*1: values provided versus Ag/AgCl (sat. KCl)

**Supplementary Table 2**: Procrustes sum of squares.

The value represents the sum of squared differences comparing the ordination results for each pair of tools. The lower the value the more similar were both NMDS results.

|  | Dalmatian plot | CyBar | CHIC | FlowFP |
| --- | --- | --- | --- | --- |
| Dalmatian plot | 0 |  |  |  |
| CyBar | 0.479 | 0 |  |  |
| CHIC | 0.345 | 0.230 | 0 |  |
| FlowFP | 0.064 | 0.040 | 0.013 | 0 |

**Supplementary Table 3**: Results for *Rr* and *So* values.

| sample | *Rr* | *So* |
| --- | --- | --- |
| 1L | 0.46 | 43.0 |
| 2L | 0.55 | 31.6 |
| 3L | 0.50 | 41.9 |
| 4L | 0.60 | 17.8 |
| 1A | 0.43 | 39.3 |
| 2A | 0.55 | 28.4 |
| 3A | 0.57 | 37.7 |
| 4A | 0.55 | 21.1 |
